# Supplementary material for: Health visiting in the UK in light of the COVID-19 pandemic experience (RReHOPE): a realist review protocol
Source: BMJ Open. 2023 Mar 8;13(3):e068544. doi: 10.1136/bmjopen-2022-068544 (PMC10008202; doi:10.1136/bmjopen-2022-068544)
Supplement: Supplementary data [file bmjopen-2022-068544supp001.pdf]

Supplementary file 1

Search strategy for Ovid MEDLINE

MEDLINE® Epub Ahead of Print, In-Process & Other Non-Indexed Citations, Ovid MEDLINE® Daily and Ovid MEDLINE® 1946 to present

1	(health visitor* or health visiting).ti,ab,kw.
2	child health program*.ti,ab,kw.
3	healthy child program*.ti,ab,kw.
4	healthy child wales.ti,ab,kw.
5	getting it right for every child.ti,ab,kw.
6	healthy child healthy future.ti,ab,kw.
7	or/1-6
8	SARS-CoV-2/ or COVID-19/ or exp COVID-19 Testing/ or exp COVID-19 Vaccines/
9	(corona* adj1 (virus* or viral*)).ti,ab,kw,kf.
10	(coronavirus* or coronoravirus* or coronaravirus* or coronovirus* or 2019nCoV* or 19nCoV* or "2019 novel*" or Ncov* or "n-cov" or "SARSCoV-2*" or "SARSCoV-2*" or SARSCoV2* or "SARS-CoV2*" or "severe acute respiratory syndrome*" or COVID*2).ti,ab,kw,kf.
11	(covid* or pandemic).ti,ab,kw,kf.
12	(CoV not (Coefficien* or "co-efficien*" or covalent* or Covington* or covariant* or covarianc* or "cut-off value*" or "cutoff value*" or "cut-off volume*" or "cutoff volume*" or "combined optimi?ation value*" or "central vessel trunk*" or CoVR or CoVS)).ti,ab,kw,kf.
13	or/8-12
14	limit 13 to yr="2020-current"
15	7 and 14

The search strategy combines general terms describing health visitors and health visiting services, and terms referring to specific UK-based programmes and policies, with terms describing the Covid-19 pandemic. The latter set of terms were adapted from existing filters developed by the UK's National Institute of Care Excellence (NICE) and Health Security Agency (HSA). Searches are limited to identify literature published from 2020 onwards.
