# Supplementary material for: Health visiting in the UK in light of the COVID-19 pandemic experience (RReHOPE): a realist review protocol
Source: BMJ Open. 2023 Mar 8;13(3):e068544. doi: 10.1136/bmjopen-2022-068544 (PMC10008202; doi:10.1136/bmjopen-2022-068544)
Supplement: Author's manuscript [file bmjopen-2022-068544.draft_revisions.pdf]

BMJ Open

BMJ Open is committed to open peer review. As part of this commitment we make the peer review history of every article we publish publicly available.

When an article is published we post the peer reviewers' comments and the authors' responses online. We also post the versions of the paper that were used during peer review. These are the versions that the peer review comments apply to.

The versions of the paper that follow are the versions that were submitted during the peer review process. They are not the versions of record or the final published versions. They should not be cited or distributed as the published version of this manuscript.

BMJ Open is an open access journal and the full, final, typeset and author-corrected version of record of the manuscript is available on our site with no access controls, subscription charges or pay-per-view fees (http://bmjopen.bmj.com).

If you have any questions on BMJ Open's open peer review process please email info.bmjopen@bmj.com

BMJ Open

Protocol: Realist Review: Health visiting in light of the COVID-19 Pandemic Experience (RReHOPE)

Journal:	*BMJ Open*
Manuscript ID	bmjopen-2022-068544
Article Type:	Protocol
Date Submitted by the Author:	23-Sep-2022
Complete List of Authors:	King, Emma; University of Stirling Faculty of Health Sciences and Sport<br>Gadsby, Erica; University of Stirling, Faculty of Health Sciences and Sport<br>Bell, Madeline; None<br>Duddy, Claire; University of Oxford, Nuffield Department of Primary Care Health Sciences<br>Kendall, Sally; University of Kent,<br>Wong, Geoff; Oxford University, Nuffield Department of Primary Care Health Sciences
Keywords:	PUBLIC HEALTH, Community child health < PAEDIATRICS, COVID-19, Quality in health care < HEALTH SERVICES ADMINISTRATION & MANAGEMENT

SCHOLARONE™
Manuscripts

Protocol: Realist Review: Health visiting in light of the COVID-19 Pandemic Experience (RReHOPE)

Corresponding Author:
Dr Emma King
FHSS, G4 Pathfoot Building
University of Stirling
Stirling, UK
FK9 4LA
emma.king@stir.ac.uk

Authors:
Emma King, Faculty of Health Science and Sport, University of Stirling, Stirling, UK.
Erica Gadsby, Faculty of Health Science and Sport, University of Stirling, Stirling, UK.
Madeline Bell, Lived Experience Coordinator (no institutional affiliation), UK.
Claire Duddy, Nuffield Department of Primary Care Health Sciences, University of Oxford, Oxford, UK.
Sally Kendall, Centre for Health Services Studies, University of Kent, Canterbury, UK.
Geoff Wong, Nuffield Department of Primary Care Health Sciences, University of Oxford, Oxford, UK.

Wordcount: 2369

Keywords:

Health visiting; Covid-19 pandemic; realist review; public health; under-fives (Infant; Infant, newborn; Child, pre-school.)

For peer review only

ABSTRACT

Introduction

Health visiting services, providing support to under-fives and their families, are organised and delivered in very different ways in different parts of the UK. Whilst there has been attention to the key components of health visiting practice and what works well and how, there is little research on how health visiting services are organised and delivered and how that affects their ability to meet their objectives. The COVID-19 pandemic rapidly disrupted service delivery from March 2020. This realist review aims to synthesise the evidence on changes during the pandemic to identify the potential for improving health visiting services and their delivery.

Methods and analysis

This review will follow the RAMESES quality standards and Pawson's five iterative stages to locate existing theories, search for evidence, select literature, extract data, synthesise evidence, and draw conclusions. It will be guided by stakeholder engagement with practitioners, commissioners, policymakers, policy advocates, and people with lived experience. This approach will consider the emerging strategies and evolving contexts in which the services are delivered, and the varied outcomes for different groups. A realist logic of analysis will be used to make sense of what was happening to health visiting services during and following the pandemic response through the identification and testing of programme theories. Our refined programme theory will then be used to develop recommendations for improving the organisation and delivery and ongoing post-pandemic recovery of health visiting services.

Ethics and dissemination

General University Ethics Panel approval has been obtained from University of Stirling (Reference 7662).

For peer review only

Dissemination will build on links to policy makers, commissioners, providers, policy advocates and the public. A range of audiences will be targeted using outputs tailored to each. A final stakeholder event focused on knowledge mobilisation will aid development of recommendations.

Registration

The review is registered with PROSPERO: 2022 CRD42022343117

Strengths and limitations of this study

- Stakeholder and lived experience input will be integral to every part of this study, including developing the focus of the review, identifying evidence, refining programme theory and 'sense-checking' recommendations.
- The review will go beyond published data to include grey literature such as reports, policy documents and other unpublished evidence.
- This realist review will follow current accepted quality and publication standards and methodological practice.
- A realist logic of analysis will be used to develop propositions that can be justified with evidence.
- This review will limit sources to those written in English and will maintain a UK-focus, so examples of early years health visiting in other countries may be excluded.

BACKGROUND

Across the four nations of the UK there is a large variability in how Child Health Programmes (CHPs) are organised and delivered.[1] CHPs offer every family an evidence-based programme of interventions and guidance to support parenting and healthy choices. Broadly, they aim to reduce inequalities and risk; ensure readiness for school; support autonomy and independence; and increase life chances and opportunity. Whilst the four UK universal CHPs are similar, there is considerable

For peer review only

variation between countries in the number and intensity of core contacts between families and services, where those contacts may take place, and who should undertake them.[2] Health visitors are specialist public health nurses who support families from the antenatal period up to school entry.[3-5] They are central to the leadership and delivery of CHPs for pre-school children.

There is little evidence to suggest how best to deliver CHPs to different groups and in different contexts. Health visiting services are complex and highly relational; partnership, integration, communication, and multi-agency work are key to service delivery.[5] We know that this service organisation and delivery affects how likely health visiting services are to succeed in their public health goals,[4, 6, 7] but we don't know much about how, or why, or in what contexts.[8]

The pandemic has provided an invaluable opportunity to reflect on the different ways that health visiting services are organized and delivered. Health visiting services were affected by the UK Government's Coronavirus Action Plan and similar plans from the devolved governments. Some critical services were not delivered and very few parents of under two's were able to see a health visitor face-to-face.[9-11] The picture varies notably between UK countries and localities, with multiple examples of differences in the local application of COVID-19 safety rules and resource prioritisation.[10, 12, 13]

There has been a proliferation of rapid studies examining COVID-related health care services in general,[14] paediatric health care,[15] and health visiting services at local and national levels.[11, 13, 16-18] The pandemic brought about rapid and significant changes to delivery of services, often with little preparation, training, or evidence of effectiveness.[11, 13] The pandemic also brought an opportunity to rethink roles, responsibilities, and service provision.[17] This opportunity can be used to understand how practitioners work effectively with families and individuals in different contexts,[19] to identify ways to improve health visiting services and to understand how to improve service recovery after the pandemic. Early child development has significant potential to affect health inequalities across the whole of society.[20, 21] The care given to a child, especially during the 1,001

For peer review only

critical days (conception to age 2), has a significant impact on the health, wellbeing, and opportunities of that child throughout life.[22]

We will use a realist review to understand how these two issues – the pandemic response and the organisation and delivery of health visiting services – impacted on each other, how, why, in what contexts, to what extent and for whom. Realist review goes beyond a standard systematic review to identify the underlying causal mechanisms of complex interventions.[23] It offers a lens through which we can make sense of what was happening to health visiting services during and following the pandemic response, particularly in understanding circumstances under which it was more or less likely to affect outcomes for children and families, through the identification and testing of programme theories.

Aim

The aim of this study is to understand the ways in which the COVID-19 pandemic has impacted on health visiting services in the UK, in order to identify how the organisation and delivery of health visiting services can be improved for a stronger post-pandemic recovery. This will be done by means of a realist review of the literature, and with key stakeholder engagement across the UK.

Review questions

i. What are the mechanisms that explain variation in and mitigation of impacts of the COVID-19 pandemic in different contexts?
ii. What are the important contexts that influence whether the different mechanisms produce the outcomes that have been identified in the literature?
iii. In what contexts are the (positive and negative) impacts likely to be most (and least) profound?
iv. What can we learn from the way health visiting services have responded to the COVID-19 pandemic to improve their organisation and delivery?

For peer review only

METHODS AND ANALYSIS

The protocol here has emerged from previous work by Gadsby et al,[15] which examined the impact of the COVID-19 pandemic response on paediatric services in Scotland and England, and by ongoing engagement with the Institute of Health Visiting's work on examining impact. Stakeholder engagement is essential in this project, given the rapidly changing context, the importance of local knowledge and unpublished materials in this review, and the complexity of differences and similarities in health visiting organisation and delivery across the UK. Approximately 25 professionals (policy leads, commissioners, practitioners, and policy advocates) will make up our stakeholder group, with representatives from Northern Ireland, Scotland, Wales, and England. Stakeholder representatives will help us to define the focus of the review, identify evidence for inclusion, refine our programme theory and 'sense-check' our recommendations.

Patient and Public involvement

In the design and drafting of this study protocol, engagement with parenting groups/networks and new parents who have encountered health visiting services during the pandemic, and insights from our patient and public involvement lead, helped to inform our realist approach and research questions. For the duration of the study, a separate group of eight people with lived experience (who have had cause to access health visiting services during the pandemic period) will work with us, alongside the professional stakeholder group. Representatives will be from Northern Ireland, Scotland, Wales and England. The involvement of both professionals and people with lived experience in the design, conduct and dissemination of this work will help to ensure this study delivers new knowledge, meaningful recommendations and accessible outputs that meet the needs of patients and the wider public, commissioners, providers and policy-makers. No patients will be directly involved as research participants in this study.

Realist review stages

For peer review only

The plan of investigation will follow this protocol, which is informed by Pawson's five iterative stages in realist reviews.[24] This process of explanation building starts with the development and refinement of a realist 'programme theory' of UK Health Visiting during the pandemic. This initial programme theory will be refined (see step 1 below) and then further refined and tested against empirical evidence during the review (see steps 2–5).

Step 1: Locate existing theories

This step entails locating underlying programme theories for health visiting service delivery during the COVID-19 pandemic, to understand how health visiting services might have been affected, in what ways, and with what consequences. Early discussions and literature scoping have informed an initial programme theory (see Figure 1). This will be further developed in step 1, in collaboration with our stakeholder groups, so that it can be tested and refined in later steps. Content experts in our professional stakeholder group and informal searches of published literature and current policy documents will help to identify existing theories. This informal searching is exploratory and aimed at quickly identifying the range of possible explanatory theories that may be relevant, using exploratory search methods, such as citation tracking and snowballing,[25] along with more structured searching for theories.[26]

Step 2: Search for evidence

To further develop and refine the programme theory from Step 1, a formal evidence search will be conducted. The search strategy will be designed, piloted and conducted by an experienced librarian (CD) in collaboration with the rest of the project team. In addition to database searches (in CINAHL, MEDLINE, Embase, HMIC and Google Scholar), relevant organisation websites will be searched, and stakeholders consulted to identify grey literature such as reports, policy documents and other unpublished evidence. 'Cited by' searches using Google Scholar, and the screening of reference lists

For peer review only

of included documents will help to ensure key references are not missed. Given the iterative nature of realist reviews, search terms will be refined, and additional searches conducted as required, to develop and test certain sub-sections of the programme theory.

Screening

Screening will be undertaken firstly against title and abstract and then by full text by the research fellow (EK). At both stages a 10% random sub-sample of the citations retrieved from searching will be reviewed independently by the co-principal investigators (co-PIs) (EG and SK) for quality control. Disagreements not resolved through discussion between the three researchers will be resolved through majority vote within the research team. Criteria for initial screening will be broad to ensure all potentially relevant evidence is included:

Inclusion:

- Type of intervention – Health Visiting Programme.
- Study design – all study designs.
- Types of settings – any setting providing Health Visiting services.
- Types of participants – all families eligible for Health Visiting services.
- Outcome measures – all outcome measures related to Health Visiting services.

Exclusion:

- Health visiting programmes run in countries other than the England, Wales, Scotland, and Northern Ireland.

For further exploratory and purposive searches, more specific inclusion and exclusion criteria will be established by the team.

Step 3: Article selection

Full text documents will be selected for inclusion in the review based on an assessment of their relevance and rigour.[27] Documents will be selected for inclusion when they contain data that could inform some aspect of the programme theory. At the point of inclusion based on relevance, an

For peer review only

assessment will also be made of rigour (how trustworthy and plausible were methods used to generate the data). Judgements about rigour will be made at the level of the included data (where necessary) and programme theory.[28] A random sample of 10% of documents will be selected and independently assessed by the co-PIs. Decisions to include/exclude will be discussed between the three researchers to ensure they have been made consistently. Any disagreements that are not resolved between the three researchers will be resolved by the team through majority vote. Any uncertainties about relevance and/or rigour in the remaining 90% will be treated in the same way: first through discussion with the co-PIs then, if necessary, resolved by the team.

Step 4: Extracting and organising data

Data extraction and organising of data will be undertaken by the research fellow (EK), with a random sample of 10% independently checked by the co-PIs (EG and SK) for quality control, with disagreements handled in the same way as above. The characteristics of the documents will be extracted for descriptive purposes into an EXCEL spreadsheet. For data analysis, the full texts of the included papers will be re-read, then uploaded into NVivo (a qualitative data analysis software tool) and initially thematically coded. This coding will be deductive (to evaluate potential propositions included in the IPT), inductive (to enable new ideas and propositions to emerge from the evidence), and retroductive (to identify and explore patterns, using theory to offer causal explanations). Refinements of codes (or theories) will be documented in attached memos. Interpretations and judgements will be subsequently confirmed with the rest of the team, and at key points, with our stakeholder groups. As refinements are made, included studies will be re-scrutinised to search for data relevant to the revised theory that may have been missed initially.

Step 5: Synthesising the evidence and drawing conclusions

A realist logic of analysis will be used to analyse the extracted data, moving between evidence and theory to develop and refine explanations about why certain patterns are occurring. Throughout the analytic process, propositions, justified with evidence, will be configured to help conceptualise underlying generative mechanisms. These propositions will be represented through Context-

For peer review only

Mechanism-Outcome Configurations (CMOCs) to describe how specific contextual factors (C) work to trigger particular mechanisms (M), to generate various outcomes (O).[29] Our stakeholder groups will help provide a richer understanding of contexts and mechanisms in different localities.

Our process of analysis and synthesis will be guided by the questions below, which will be asked in relation to each data source.[30]

- Relevance: Are sections of text relevant to programme theory development?
- Rigour: Are these data sufficiently trustworthy to warrant making changes to any aspect of the programme theory?
- Interpretation of meaning: If the section of text is relevant and trustworthy enough, do its contents provide data that may be interpreted as functioning as context, mechanism or outcome?
- Interpretations and judgements about CMOCs: What is the CMOC (partial or complete) for the data that has been interpreted as functioning as context, mechanism or outcome? Are there further data to inform the particular CMOCs contained within this document or other documents? If so, which other documents? How does this particular CMOC relate to other CMOCs that have already been developed?
- Interpretations and judgements about programme theory: How does this particular (full or partial) CMOC relate to the programme theory? Within this same document are there data which inform how the CMOC relates to the programme theory? If not, are there data in other documents? Which ones? In light of this particular CMOC and any supporting data, does the programme theory need to be changed?

Not all parts of a CMOC configuration will be present in the same document, so synthesising data from different documents will be necessary to inform our interpretation of the relationships between contexts, mechanisms and outcomes.

When working through the questions set out, where appropriate we will use the following forms of reasoning to make sense of the data:

For peer review only

- Juxtaposition of data: for example, where data about how the pandemic influenced outcomes in the universal health visiting programme in one document enables insights into data about outcomes in another document.
- Reconciling of data: where data differ in apparently similar circumstances, further investigation is appropriate in order to find explanations for why these differences have occurred.
- Adjudication of data: on the basis of methodological strengths or weaknesses.
- Consolidation of data: where outcomes differ in particular contexts, an explanation can be constructed of how and why these outcomes occur differently.

DISCUSSION

Our programme theory will be developed through engagement with stakeholders and those with lived experience and grounded in what the pandemic has taught us about how to better deliver health visiting services across the UK. Our programme theory will be used to develop recommendations for improving the organisation and delivery of health visiting services for different groups and in different settings.

To recommend improvement we will use our realist review to identify the most important mechanisms with the programme theories and how these can be 'triggered' to achieve the desired outcomes. We will also identify which contexts are related to these key mechanisms and suggest what strategies might be used to change the contexts in such a way that these key mechanisms are triggered to produce the desired outcomes.

For peer review only

DISSEMINATION AND ETHICS

Dissemination

We will discuss emerging findings and 'sense-check' and further refine recommendations with our stakeholder groups. Our dissemination strategy will build on an integrated approach with input from key stakeholders and particularly the Institute of Health Visiting. We will target outputs at a range of audiences, including policy makers and commissioners of Health Visiting services, providers of Health Visiting and early years services, and members of the public.

Ethics

General University Ethics Panel approval has been obtained from University of Stirling (Reference 7662).

FUNDING STATEMENT

This study is funded by the NIHR Health and Social Care Delivery Research Programme NIHR134986. The views expressed are those of the author(s) and not necessarily those of the NIHR or the Department of Health and Social Care.

ACKNOWLEDGEMENTS

We wish to thank the members of our stakeholder and lived experience groups for their ongoing involvement in this study.

AUTHORS' CONTRIBUTIONS

Emma King – Methodology, Writing - Original Draft, Writing - Review & Editing,

Erica Gadsby – Conceptualisation, Methodology, Funding Acquisition, Supervision Writing - Review & Editing

For peer review only

Madeline Bell - Conceptualisation, Lived Experience Engagement, Writing - Review & Editing

Claire Duddy - Conceptualisation, Methodology, Funding Acquisition, Writing - Original Draft, Data Curation Writing - Review & Editing

Sally Kendall - Conceptualisation, Methodology, Funding Acquisition, Writing - Review & Editing

Geoff Wong - Conceptualisation, Methodology, Funding Acquisition, Writing - Review & Editing

COMPETING INTERESTS

None declared

FIGURES

Figure 1: Initial Programme Theory with possible contexts, mechanisms and outcomes of interest

peer review only

REFERENCES

1. Hackett A, Clarke K, Wilkinson J. Social Policy, Context of Public Health and Organisation of Health Visiting Provision. In: Burrow P, Cowie J, editors. Health Visiting: Specialist Community Public Health Nursing 3rd ed Great Britain: Elsevier; 2022.
2. Whittaker K, Appleton, J.V., Peckover, S. and Adams, C. . Organising health visiting services in the UK: Frontline perspectives. . Journal of Health Visiting. 2021;9(2):68-75.
3. First 1001 Days Movement. Why Health Visitors Matter: Perspectives on a widely valued service. https://parentinfantfoundation.org.uk/wp-content/uploads/2022/05/Why-Health-Visitors-Matter.pdf; 2022.
4. Cowley S, Whittaker K, Malone M, Donetto S, Grigulis A, Maben J. Why health visiting? Examining the potential public health benefits from health visiting practice within a universal service: A narrative review of the literature. International Journal of Nursing Studies. 2015;52(1):465-80.
5. Public Health England. Health visiting and school nursing service delivery model (updated 19 May 2021). Available: https://www.gov.uk/government/publications/commissioning-of-public-health-services-for-children/health-visiting-and-school-nursing-service-delivery-model (17/05/22); 2021.
6. Axford N, Barlow, J., Coad, J., Schrader-Mcmillan, A., Bjornstad, G., Wrigley, Z., Goodwin, A., Ohlson, C., Sonthalia, S., Toft, A., Whybra, L. and Wilkinson, T. . Rapid Review to Update Evidence for the Healthy Child Programme 0–5. Available: https://assets.publishing.service.gov.uk/government/uploads/system/uploads/attachment_data/file/429740/150520RapidReviewHealthyChildProg_UPDATE_poisons_final.pdf (17/05/22); 2015.
7. Cowley S, Whittaker K, Malone M, Donetto S, Grigulis A, Maben J. What makes health visiting successful—or not? 1. Universality. Journal of Health Visiting. 2018;6(7):352-60.
8. Bunn F, Kendall S. Does nursing research impact on policy? A case study of health visiting research and UK health policy. Journal of Research in Nursing. 2011;16(2):169-91.
9. Conti G, Dow A. The impacts of COVID-19 on Health Visiting in England: First Results. Available: https://discovery.ucl.ac.uk/id/eprint/10106430 (17/05/22); 2020a.
10. Conti G, Dow A. The impacts of COVID-19 on Health Visiting Services in England: FOI Evidence for the First Wave. Available: https://discovery.ucl.ac.uk/id/eprint/10122752 (17/05/22); 2020b.
11. Saunders B, Hogg S. Babies in Lockdown: listening to parents to build back better. . Available: https://parentinfantfoundation.org.uk/our-work/campaigning/babies-in-lockdown/#fullreport (17/05/22); 2020.
12. Institute of Health Visiting. Making history: Health visiting during COVID-19. Available: https://ihv.org.uk/wp-content/uploads/2021/07/Health-visiting-making-history-case-studies-FINAL-VERSION-updated-14.7.21.pdf (17/05/22); 2020b.
13. Reed J, Parish N. Working for babies: Lockdown lessons from local systems. Available: https://parentinfantfoundation.org.uk/wp-content/uploads/2021/01/210115-F1001D-Working-for-Babies-Report-FINAL-v1.1-compressed-2.pdf (17/05/22); 2021.
14. Lewis R, Pereira, P., Thorlby, R. and Warburton, W. . Understanding and sustaining the health care service shifts accelerated by COVID-19. 2020.
15. Gadsby EW, Christie-de Jong, F., Bhopal, S., Corlett, H. and Turner, S. . Qualitative analysis of the impact of the SARS-CoV-2 pandemic response on paediatric health services in North of Scotland and North of England. BMJ Open. 2022;12(2).

For peer review only

16. Barlow J, Bach-Mortensen, A., Homonchuk, O. and Woodman, J. The impact of the COVID-19 pandemic on services from pregnancy through age 5 years for families who are high risk of poor outcomes or who have complex social needs. 2020.
17. Bynner C, McBride M. Children's Neighbourhood Scotland - Early insights into the COVID-19 response: 8 May 2020. . Available: http://eprints.gla.ac.uk/215811 (17/05/22). 2020.
18. Morton A. Health visiting during COVID-19 and beyond: the impact on children and families. Local Government Association webinar: Supporting the development of babies and young children during the COVID-19 outbreak, ; 2020.
19. Bryans A, Cornish, F. and McIntosh, J. . The potential of ecological theory for building an integrated framework to develop the public health contribution of health visiting. Health & Social Care in the Community. 2009;17(6):564-72.
20. Black MM, Walker, S.P., Fernald, L.C.H., Andersen, C.T., Digirolamo, A.M., Lu, C., Mccoy, D.C., Fink, G., Shawar, Y.R., Shiffman, P.J., Devercelli, A.E., Wodon, Q.T., Vargas-Barón, E. and Grantham-Mcgregor, S. . Advancing Early Childhood Development: from Science to Scale 1: Early childhood development coming of age: science through the life course. . The Lancet (British Edition). 2016;389(10064):77-90.
21. National Research C, Institute of Medicine Committee on Integrating the Science of Early Childhood D. In: Shonkoff JP, Phillips DA, editors. From Neurons to Neighborhoods: The Science of Early Childhood Development. Washington (DC): National Academies Press (US)

Copyright 2000 by the National Academy of Sciences. All rights reserved.; 2000.
22. HM Government. The Best Start for Life: A Vision for the 1,001 Critical Days. The Early Years Healthy Development Review Report CP419 (25 March 2021) Available: https://www.gov.uk/government/publications/the-best-start-for-life-a-vision-for-the-1001-critical-days (17/05/22); 2021.
23. Berg RC, Nanavati J. Realist Review: Current Practice and Future Prospects. Journal of Research Practice. 2016;12:1.
24. Pawson R, Greenhalgh, T., Harvey, G. and Walshe, K. Realist synthesis - an introduction. London: ESRC; 2004.
25. Greenhalgh T, Peacock R. Effectiveness and efficiency of search methods in systematic reviews of complex evidence: audit of primary sources. BMJ. 2005;331(7524):1064-5.
26. Booth A, Clarke, M., Ghersi, D., Moher, D., Petticrew, M. and Stewart, L. . An international registry of systematic-review protocols. The Lancet (British Edition). 2011;377(9760):108-9.
27. Pawson R. Evidence-based policy: a realist perspective. London: Sage; 2011.
28. Wong G. Data gathering for realist reviews: Looking for needles in haystacks. In: Emmel N, Greenhalgh J, Manzano A, Monaghan M, Dalkin S, editors. Doing Realist Research. London: SAGE; 2018.
29. Pawson R, Tilley N. An introduction to scientific realist evaluation. Evaluation for the 21st century: A handbook: Sage Publications, Inc; 1997. p. 405-18.
30. Papoutsi C, Mattick, K., Pearson, M., Brennan, N., Briscoe, S. and Wong, G. Interventions to improve antimicrobial prescribing of doctors in training (IMPACT): a realist review. Health Services and Delivery Research. 2018;6(10).

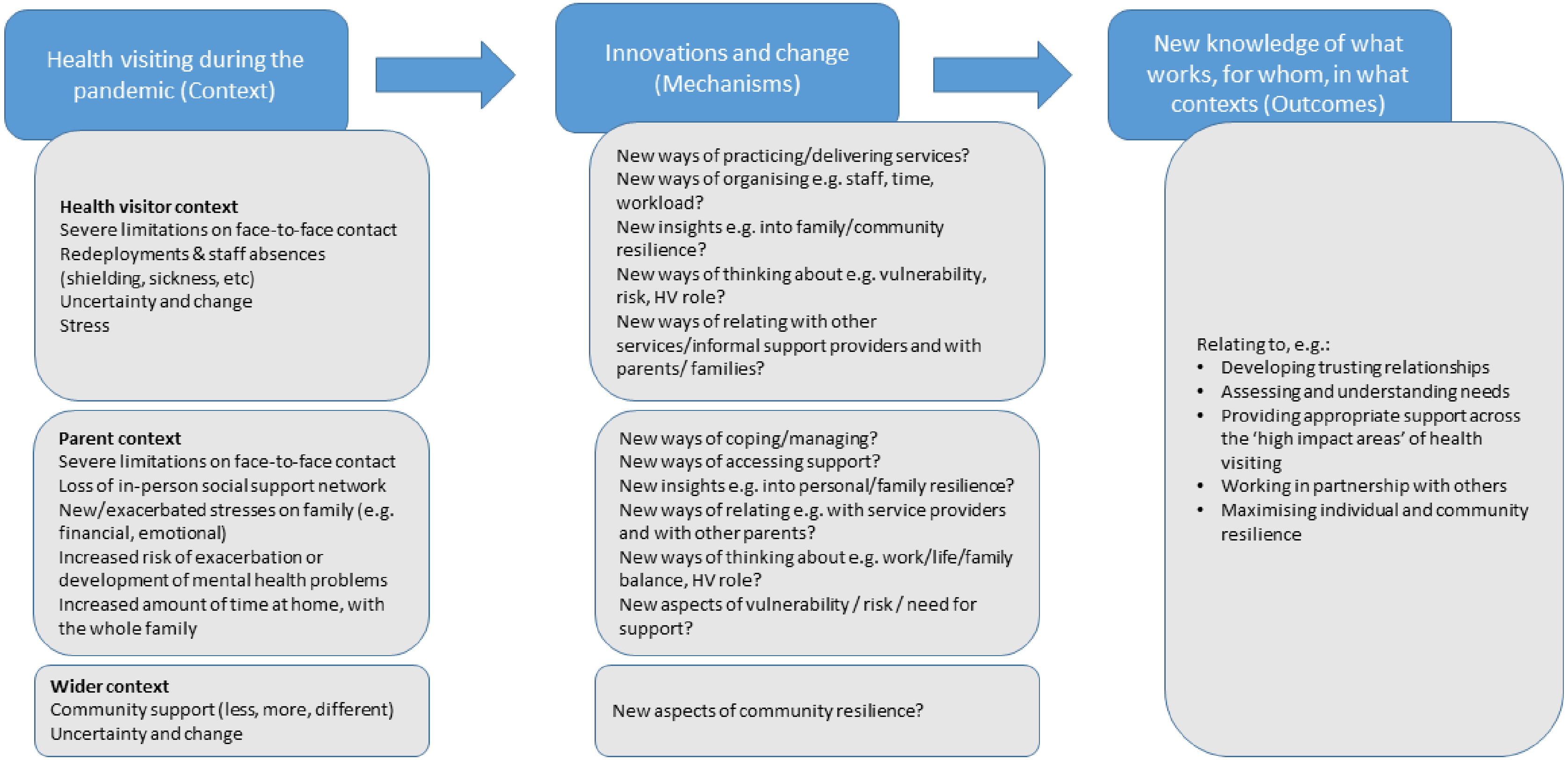
Health visiting during the pandemic (Context)
Innovations and change (Mechanisms)
New knowledge of what works, for whom, in what contexts (Outcomes)
Health visitor context
Severe limitations on face-to-face contact
Redeployments & staff absences (shielding, sickness, etc)
Uncertainty and change
Stress
New ways of practicing/delivering services?
New ways of organising e.g. staff, time, workload?
New insights e.g. into family/community resilience?
New ways of thinking about e.g. vulnerability, risk, HV role?
New ways of relating with other services/informal support providers and with parents/ families?
Parent context
Severe limitations on face-to-face contact
Loss of in-person social support network
New/exacerbated stresses on family (e.g. financial, emotional)
Increased risk of exacerbation or development of mental health problems
Increased amount of time at home, with the whole family
New ways of coping/managing?
New ways of accessing support?
New insights e.g. into personal/family resilience?
New ways of relating e.g. with service providers and with other parents?
New ways of thinking about e.g. work/life/family balance, HV role?
New aspects of vulnerability / risk / need for support?
Wider context
Community support (less, more, different)
Uncertainty and change
New aspects of community resilience?
Relating to, e.g.:
• Developing trusting relationships
• Assessing and understanding needs
• Providing appropriate support across the 'high impact areas' of health visiting
• Working in partnership with others
• Maximising individual and community resilience

RAMESES II reporting standards for realist evaluations*

Protocol: Realist Review: Health visiting in light of the COVID-19 Pandemic Experience (RReHOPE)

	Reported in Document	Pages in Document
TITLE		
1. In the title, identify the document as a realist evaluation.	Y	1
SUMMARY OR ABSTRACT		
2. Journal articles will usually require an abstract, while reports and other forms of publication will usually benefit from a short summary. The abstract or summary should include brief details on: the policy, programme or initiative under evaluation; programme setting; purpose of the evaluation; evaluation question(s) and/or objective(s); evaluation strategy; data collection, documentation and analysis methods; key findings and conclusions Where journals require it and the nature of the study is appropriate, brief details of respondents to the evaluation and recruitment and sampling processes may also be included Sufficient detail should be provided to identify that a realist approach was used and that realist programme theory was developed and/or refined.	Y	1,2
INTRODUCTION		
3. Rationale for evaluation. Explain the purpose of the evaluation and the implications for its focus and design.	Y	3
4. Programme theory. Describe the initial programme theory (or theories) that underpin the programme, policy or initiative.	Y	4
5. Evaluation questions, objectives and focus. State the evaluation question(s) and specify the objectives for the evaluation. Describe whether and how the programme theory was used to define the scope and focus of the evaluation.	Y	3,4
6. Ethical approval. State whether the realist evaluation required and has gained ethical approval from the relevant authorities, providing details as appropriate. If ethical approval was deemed unnecessary, explain why.	Y	1
METHODS		
7. Rationale for using realist evaluation Explain why a realist evaluation approach was chosen and (if relevant) adapted.	Y	3
8. Environment surrounding the evaluation. Describe the environment in which the evaluation took place.	Y	2,3
9. Describe the programme policy, initiative or product evaluated. Provide relevant details on the programme, policy or initiative evaluated.	Y	2,3
10. Describe and justify the evaluation design. A description and justification of the evaluation design (i.e. the account of what was planned, done and why) should be included, at least in summary form or as an appendix, in the document which presents the main findings. If this is not done, the omission should be justified and a reference or link to the evaluation design given. It may also be useful to publish or make freely available (e.g. online on a website) any original evaluation design document or protocol, where they exist.	Y	4-6
11. Data collection methods. Describe and justify the data collection methods – which ones were used, why and how they fed into developing, supporting, refuting or refining programme theory. Provide details of the steps taken to enhance the trustworthiness of data collection and documentation.	Y	6
12. Recruitment process and sampling strategy. Describe how respondents to the evaluation were recruited or engaged and how the sample contributed to the development, support, refutation or refinement of programme theory.	N	n/a
13. Data analysis. Describe in detail how data were analysed. This section should include information on the constructs that were identified, the process of analysis, how the programme theory was further developed, supported, refuted and refined, and (where relevant) how analysis changed as the evaluation unfolded.	N	n/a
RESULTS		
14. Details of participants. Report (if applicable) who took part in the evaluation, the details of the data they provided and how the data was used to develop, support, refute or refine programme theory.	N	n/a
15. Main findings. Present the key findings, linking them to contexts, mechanisms and outcome configurations. Show how they were used to further develop, test or refine the programme theory.	N	n/a
DISCUSSION		
16. Summary of findings. Summarise the main findings with attention to the evaluation questions, purpose of the evaluation, programme theory and intended audience.	N	n/a
17. Strengths, limitations and future directions Discuss both the strengths of the evaluation and its limitations. These should include (but need not be limited to): (1) consideration of all the steps in the evaluation processes; and (2) comment on the adequacy, trustworthiness and value of the explanatory insights which emerged. In many evaluations, there will be an expectation to provide guidance on future directions for the programme, policy or initiative, its implementation and/or design. The particular implications arising from the realist nature of the findings should be reflected in these discussions	PARTLY	1, 7
18. Comparison with existing literature. Where appropriate, compare and contrast the evaluation's findings with the existing literature on similar programmes, policies or initiatives.	N	n/a
19. Conclusion and recommendations. List the main conclusions that are justified by the analyses of the data. If appropriate, offer recommendations consistent with a realist approach.	N	n/a
20. Funding and conflict of interest. State the funding source (if any) for the evaluation, the role played by the funder (if any) and any conflicts of interests.	Y	7

*Wong, G., Westhorp, G., Manzano, A. *et al.* RAMESES II reporting standards for realist evaluations. *BMC Med* **14**, 96 (2016). https://doi.org/10.1186/s12916-016-0643-1

For peer review only

Health visiting in light of the COVID-19 Pandemic Experience (RReHOPE): A realist review protocol

Journal:	*BMJ Open*
Manuscript ID	bmjopen-2022-068544.R1
Article Type:	Protocol
Date Submitted by the Author:	08-Feb-2023
Complete List of Authors:	King, Emma; University of Stirling Faculty of Health Sciences and Sport<br>Gadsby, Erica; University of Stirling, Faculty of Health Sciences and Sport<br>Bell, Madeline; None<br>Duddy, Claire; University of Oxford, Nuffield Department of Primary Care Health Sciences<br>Kendall, Sally; University of Kent,<br>Wong, Geoff; Oxford University, Nuffield Department of Primary Care Health Sciences
**Primary Subject Heading**:	Paediatrics
Secondary Subject Heading:	Nursing, Public health
Keywords:	PUBLIC HEALTH, Community child health < PAEDIATRICS, COVID-19, Quality in health care < HEALTH SERVICES ADMINISTRATION & MANAGEMENT

SCHOLARONE™
Manuscripts

Health visiting in light of the COVID-19 Pandemic Experience (RReHOPE): A realist review protocol

Corresponding Author:
Dr Emma King
FHSS, G4 Pathfoot Building
University of Stirling
Stirling, UK
FK9 4LA
emma.king@stir.ac.uk

Authors:
Emma King, Faculty of Health Science and Sport, University of Stirling, Stirling, UK.
Erica Gadsby, Faculty of Health Science and Sport, University of Stirling, Stirling, UK.
Madeline Bell, Lived Experience Coordinator (no institutional affiliation), UK.
Claire Duddy, Nuffield Department of Primary Care Health Sciences, University of Oxford, Oxford, UK.
Sally Kendall, Centre for Health Services Studies, University of Kent, Canterbury, UK.
Geoff Wong, Nuffield Department of Primary Care Health Sciences, University of Oxford, Oxford, UK.

Wordcount: 3144

Keywords:

Health visiting; COVID-19 pandemic; realist review; public health; under-fives (Infant; Infant, newborn; Child, pre-school.)

For peer review only

ABSTRACT

Introduction

Health visiting services, providing support to under-fives and their families, are organised and delivered in very different ways in different parts of the UK. Whilst there has been attention to the key components of health visiting practice and what works well and how, there is little research on

how health visiting services are organised and delivered and how that affects their ability to meet their objectives. The COVID-19 pandemic rapidly disrupted service delivery from March 2020. This realist review aims to synthesise the evidence on changes during the pandemic to identify the potential for improving health visiting services and their delivery.

Methods and analysis

This review will follow the RAMESES quality standards and Pawson's five iterative stages to locate existing theories, search for evidence, select literature, extract data, synthesise evidence, and draw conclusions. It will be guided by stakeholder engagement with practitioners, commissioners, policymakers, policy advocates, and people with lived experience. This approach will consider the emerging strategies and evolving contexts in which the services are delivered, and the varied outcomes for different groups. A realist logic of analysis will be used to make sense of what was happening to health visiting services during and following the pandemic response through the identification and testing of programme theories. Our refined programme theory will then be used to develop recommendations for improving the organisation and delivery and ongoing post-pandemic recovery of health visiting services.

Ethics and dissemination

General University Ethics Panel approval has been obtained from University of Stirling (Reference 7662).

Dissemination will build on links to policy makers, commissioners, providers, policy advocates and the public. A range of audiences will be targeted using outputs tailored to each. A final stakeholder event focused on knowledge mobilisation will aid development of recommendations.

Registration

The review is registered with PROSPERO: 2022 CRD42022343117

For peer review only

Strengths and limitations of this study

- Stakeholder and lived experience input will be integral to every part of this study, including developing the focus of the review, identifying evidence, refining programme theory and 'sense-checking' recommendations.
- The review will go beyond published data to include grey literature such as reports, policy documents and other unpublished evidence.
- This realist review will follow current accepted quality and publication standards and methodological practice.
- A realist logic of analysis will be used to develop propositions that can be justified with evidence.
- This review will limit sources to those written in English and will maintain a UK-focus, so examples of early years health visiting in other countries may be excluded.

BACKGROUND

Child Health Programmes (CHPs) in the UK are universal in reach and offer every family an evidence-based programme of interventions and guidance to support parenting and healthy choices. Broadly, they aim to reduce inequalities and risk; ensure readiness for school; support autonomy and independence; and increase life chances and opportunity.[1] The early years element (0 to 5) of CHPs is led by Health Visitors – registered nurses/midwives with additional training in community public health nursing. Health Visiting teams include a variety of practitioners, such as community staff nurses and nursery nurses, and link closely with school nurses who lead the 5 to 19 element of the programme. Whilst the objectives of the universal CHPs across the four nations of the UK are similar, there is considerable variation in how they are organised and delivered.[2] For example, there is variation in the number and intensity of core contacts between families and services, where those contacts may take place, and who should undertake them.[3] Whilst models of health visiting can be

For peer review only

found elsewhere, the policy contexts influencing service commissioning, delivery, organisation and nursing/professional education differ significantly across countries.[4]

There is little evidence to suggest how best to deliver CHPs to different groups and in different contexts. Health visiting services are complex and highly relational; partnership, integration, communication, and multi-agency work are key to service delivery.[5] We know that this service organisation and delivery affects how likely health visiting services are to succeed in their public health goals,[6-8] but we don't know much about how, or why, or in what contexts.[9]

The pandemic has provided an invaluable opportunity to reflect on the different ways that health visiting services are organized and delivered. Health visiting services were affected by the UK Government's Coronavirus Action Plan and similar plans from the devolved governments. Some critical services were not delivered and very few parents of under two's were able to see a health visitor face-to-face.[10-12] The picture varies notably between UK countries and localities, with multiple examples of differences in the local application of COVID-19 safety rules and resource prioritisation.[11, 13, 14]

What happens in health visiting has sometimes been referred to as a 'black box' of complexities. [15-17] Whilst there is undoubted value in health visiting, 'the what and the how' (what happens inside that black box) is less clear. The COVID-19 pandemic might be conceptualised as a natural experiment which forced rapid and dramatic changes to the way health visiting was delivered. From this, new data is emerging that might help to reveal explanations of why, how, and when certain outcomes occur. This presents an opportunity, therefore, to learn new things about the service in relation to what works, for whom in what circumstances.

There has been a proliferation of rapid studies examining COVID-related health care services in general,[18] paediatric health care,[19] and health visiting services at local and national levels.[12, 14, 20-22] The pandemic brought about rapid and significant changes to delivery of services, often with little preparation, training, or evidence of effectiveness.[12, 14] The pandemic also brought an opportunity to rethink roles, responsibilities, and service provision.[21] These changes can be

For peer review only

investigated to better understand how practitioners work effectively with families and individuals in different contexts,[23] to identify ways to improve health visiting services, and to understand how to improve service recovery after the pandemic. Early child development has significant potential to affect health inequalities across the whole of society.[24, 25] The care given to a child, especially during the 1,001 critical days (conception to age 2), has a significant impact on the health, wellbeing, and opportunities of that child throughout life.[26]

We will use a realist review to understand how these two issues – the pandemic response and the organisation and delivery of UK health visiting services – impacted on each other, how, why, in what contexts, to what extent and for whom. Realist review goes beyond a standard systematic review to identify the underlying causal mechanisms of complex interventions.[27] Within realist reviews, mechanisms are conceptualised as hidden, context-sensitive, causal forces. Mechanisms may be found at different 'levels' (e.g. organisation or individual level) but to make the claim that something is functioning as a mechanism, it is important to consider for which outcome this claim is being made. Importantly, mechanisms are not merely an intervention, an activity, or an intervention component [28, 29]. Hence, realist reviews will produce knowledge claims about causes of outcomes in certain contexts using the Context-Mechanisms-Outcome configuration heuristic, as this links the three main concepts of a realist analysis.

This realist review approach will help to make sense of what was happening to health visiting services during and following the pandemic response, particularly in understanding circumstances under which it was more or less likely to affect outcomes for children and families, through the identification and testing of programme theories. The rapidly changing context during COVID will allow us to investigate the programmatic assumptions underlying health visiting, because so many of these accepted ways of working were changed during the COVID-19 pandemic response. From this, we anticipate recommendations for service improvement to emerge.

Aim

For peer review only

The aim of this study is to understand the ways in which the COVID-19 pandemic has impacted on health visiting services in the UK, in order to identify how the organisation and delivery of health visiting services can be improved for a stronger post-pandemic recovery in service delivery. This will be done by means of a realist review of the literature, and with key stakeholder engagement across the UK.

The study seeks to answer the question: How can the organisation and delivery of health visiting services in the UK be improved in light of the COVID-19 pandemic, to provide equitable, effective and efficient services for young children and their families?

Objectives

1) To conduct a realist review of the literature to examine what the impacts (both positive and negative) of the COVID-19 pandemic have been on health visiting services in the UK, for whom, in different contexts.

2) To engage with key policy, practice and research stakeholders in England, Scotland, Wales and Northern Ireland to understand important contextual differences across the UK in relation to the planning, organisation and delivery of health visiting services.

3) To identify recommendations for improving the organisation and delivery and ongoing post-pandemic recovery of health visiting services in different settings, for different groups.

The project will run from 1st June 2022 to 30th November 2023.

METHODS AND ANALYSIS

The protocol here has emerged from previous work by Gadsby et al,[19] which examined the impact of the COVID-19 pandemic response on paediatric services in Scotland and England, and by ongoing engagement with the Institute of Health Visiting's work on examining impact. Stakeholder

For peer review only

engagement is essential in this project, given the rapidly changing context, the importance of local knowledge and unpublished materials in this review, and the complexity of differences and similarities in health visiting organisation and delivery across the UK. Approximately 25 professionals (policy leads, commissioners, practitioners, and policy advocates) will make up our stakeholder group, with representatives from Northern Ireland, Scotland, Wales, and England. This number is manageable from a practical point of view, yet will ensure we can include appropriate expertise by mapping stakeholders onto a grid of five professional areas (policy, commissioning, practice, academia, and advocacy) covering five geographical areas (Northern Ireland, Scotland, Wales, England, and UK wide).[30] Stakeholders will be approached through targeted sampling and snowball sampling. This group will meet six times throughout the 18 months of the study. Stakeholder representatives will help us to define the focus of the review, identify evidence for inclusion, refine our programme theory and 'sense-check' our recommendations.

Patient and Public involvement

In the design and drafting of this study protocol, engagement with parenting groups/networks and new parents who have encountered health visiting services during the pandemic, and insights from our patient and public involvement lead, helped to inform our realist approach and research questions. For the duration of the study, a separate group of eight people with lived experience (who have had cause to access health visiting services during the pandemic period) will work with us, alongside the professional stakeholder group. Our target of eight lived experience representatives was chosen to allow two representatives from each of the different countries (Northern Ireland, Scotland, Wales, and England) whilst still being a small enough group to allow for meaningful discussion and contribution during online meetings. Potential representatives will be sought through advertising on social media and press releases. Where possible, we will purposively select members of the group to achieve a mixed group profile, for example based on where they live, how many children they look after and ethnicity. This group will meet four times throughout the 18 months of the study.

For peer review only

The involvement of both professionals and people with lived experience in the design, conduct and dissemination of this work will help to ensure this study delivers new knowledge, meaningful recommendations and accessible outputs that meet the needs of patients and the wider public, commissioners, providers and policy-makers. No patients will be directly involved as research participants in this study.

Realist review stages

The plan of investigation will follow this protocol, which is informed by Pawson's five iterative stages in realist reviews.[31] This process of explanation building starts with the development and refinement of a realist 'programme theory' of UK Health Visiting during the pandemic. This initial programme theory will be refined (see step 1 below) and then further refined and tested against empirical evidence during the review (see steps 2–5).

Step 1: Locate existing theories

This step entails locating underlying programme theories for health visiting service delivery during the COVID-19 pandemic, to understand how health visiting services might have been affected, in what ways, and with what consequences. Early discussions and literature scoping have informed an initial programme theory (see Figure 1), showing the possible contexts, mechanisms, and outcomes of interest. If we assume that the mechanisms by which health visiting outcomes are achieved take effect in certain activating contexts, then the changes in service organisation and delivery prompted by the pandemic might well reveal new understandings of these contexts and mechanisms. The changes in context may reveal explanations of why, how, and when certain outcomes occur.

This will be further developed in step 1, in collaboration with our stakeholder groups, so that it can be tested and refined in later steps. Content experts in our professional stakeholder group and informal searches of published literature and current policy documents will help to identify existing theories. This informal searching is exploratory and aimed at quickly identifying the range of possible

For peer review only

explanatory theories that may be relevant, using exploratory search methods, such as citation tracking and snowballing,[32] along with more structured searching for theories.[33]

Figure 1 here

Step 2: Search for evidence

To further develop and refine the programme theory from Step 1, a formal evidence search will be conducted. The search strategy will combine general terms describing health visitors and health visiting services, and terms referring to specific UK-based programmes and policies, with terms describing the COVID-19 pandemic. (Supplementary File 1) Searches will be limited to identify literature published from 2020 onwards (to coincide with the start of the COVID-19 pandemic response in the UK).

The search strategy will be designed, piloted and conducted by an experienced librarian (CD) in collaboration with the rest of the project team. In addition to database searches (in CINAHL, MEDLINE, Embase, HMIC and Google Scholar), relevant organisation websites will be searched, and stakeholders consulted to identify grey literature such as reports, policy documents and other unpublished evidence. 'Cited by' searches using Google Scholar, and the screening of reference lists of included documents will help to ensure key references are not missed. Given the iterative nature of realist reviews, search terms will be refined, and additional searches conducted as required, to develop and test certain sub-sections of the programme theory.

For peer review only

Screening

Screening will be undertaken firstly against title and abstract and then by full text by the research fellow (EK). At both stages a 10% random sub-sample of the citations retrieved from searching will be reviewed independently by the co-principal investigators (co-PIs) (EG and SK) for quality control. Disagreements not resolved through discussion between the three researchers will be resolved

through majority vote within the research team. Criteria for initial screening will be broad to ensure all potentially relevant evidence is included:

Inclusion:

- Type of intervention – Health Visiting Programme.
- Study design – all study designs.
- Types of settings – any setting providing Health Visiting services.
- Types of participants – all families eligible for Health Visiting services.
- Outcome measures – all outcome measures related to Health Visiting services.

Exclusion:

- Health visiting type models or programmes run in countries other than the England, Wales, Scotland, and Northern Ireland.

For further exploratory and purposive searches, more specific inclusion and exclusion criteria will be established by the team. This is what Booth has termed the 'mushroom' method of searching, combining an overall search (the mushroom cap) with additional specific search(es) (the mushroom stalk). [34] This review looks specifically at the UK health system, to best inform the delivery of UK CHPs within their specific political and historical contexts. We may expand searches to include literature on services closely aligned with health visiting, e.g. police or social work. We may also expand our criteria to look at similar roles in other countries, although health visiting in its practice orientation to prevention and health promotion for the 0-5 year age group is rarely observed in other countries despite the provision of other child health nursing models.[4]

Step 3: Article selection

Full text documents will be selected for inclusion in the review based on an assessment of their relevance and rigour.[35] Documents will be selected for inclusion when they contain data that could inform some aspect of the programme theory. At the point of inclusion based on relevance, an assessment will also be made of rigour (how trustworthy and plausible were methods used to

For peer review only

generate the data). Judgements about rigour will be made at the level of the included data (where necessary) and programme theory.[36] A random sample of 10% of documents will be selected and independently assessed by the co-PIs. Decisions to include/exclude will be discussed between the three researchers to ensure they have been made consistently. Any disagreements that are not resolved between the three researchers will be resolved by the team through majority vote. Any uncertainties about relevance and/or rigour in the remaining 90% will be treated in the same way: first through discussion with the co-PIs then, if necessary, resolved by the team.

Step 4: Extracting and organising data

Data extraction and organising of data will be undertaken by the research fellow (EK), with a random sample of 10% independently checked by the co-PIs (EG and SK) for quality control, with disagreements handled in the same way as above. The characteristics of the documents will be extracted for descriptive purposes into an EXCEL spreadsheet. For data analysis, the full texts of the included papers will be re-read, then uploaded into NVivo (a qualitative data analysis software tool) and initially thematically coded. This coding will be deductive (to evaluate potential propositions included in the IPT), inductive (to enable new ideas and propositions to emerge from the evidence), and retroductive (to identify and explore patterns, using theory to offer causal explanations). Refinements of codes (or theories) will be documented in attached memos. Interpretations and judgements will be subsequently confirmed with the rest of the team, and at key points, with our stakeholder groups. As refinements are made, included studies will be re-scrutinised to search for data relevant to the revised theory that may have been missed initially.

Step 5: Synthesising the evidence and drawing conclusions

A realist logic of analysis will be used to analyse the extracted data, moving between evidence and theory to develop and refine explanations about why certain patterns are occurring. Throughout the analytic process, propositions, justified with evidence, will be configured to help conceptualise underlying generative mechanisms. These propositions will be represented through Context-Mechanism-Outcome Configurations (CMOCs) to describe how specific contextual factors (C) work to

For peer review only

trigger particular mechanisms (M), to generate various outcomes (O).[37] Our stakeholder groups will help provide a richer understanding of contexts and mechanisms in different localities.

Our process of analysis and synthesis will be guided by the questions below, which will be asked in relation to each data source.[38]

- Relevance: Are sections of text relevant to programme theory development?
- Rigour: Are these data sufficiently trustworthy to warrant making changes to any aspect of the programme theory?
- Interpretation of meaning: If the section of text is relevant and trustworthy enough, do its contents provide data that may be interpreted as functioning as context, mechanism or outcome?
- Interpretations and judgements about CMOCs: What is the CMOC (partial or complete) for the data that has been interpreted as functioning as context, mechanism or outcome? Are there further data to inform the particular CMOCs contained within this document or other documents? If so, which other documents? How does this particular CMOC relate to other CMOCs that have already been developed?
- Interpretations and judgements about programme theory: How does this particular (full or partial) CMOC relate to the programme theory? Within this same document are there data which inform how the CMOC relates to the programme theory? If not, are there data in other documents? Which ones? In light of this particular CMOC and any supporting data, does the programme theory need to be changed?

Not all parts of a CMOC configuration will be present in the same document, so synthesising data from different documents will be necessary to inform our interpretation of the relationships between contexts, mechanisms and outcomes.

When working through the questions set out, where appropriate we will use the following forms of reasoning to make sense of the data:

For peer review only

- Juxtaposition of data: for example, where data about how the pandemic influenced outcomes in the universal health visiting programme in one document enables insights into data about outcomes in another document.
- Reconciling of data: where data differ in apparently similar circumstances, further investigation is appropriate in order to find explanations for why these differences have occurred.
- Adjudication of data: on the basis of methodological strengths or weaknesses.
- Consolidation of data: where outcomes differ in particular contexts, an explanation can be constructed of how and why these outcomes occur differently.

DISSEMINATION AND ETHICS

Dissemination

We will discuss emerging findings and ‘sense-check’ and further refine recommendations with our stakeholder groups. Our dissemination strategy will build on an integrated approach with input from key stakeholders and particularly the Institute of Health Visiting. We will target outputs at a range of audiences, including policy makers and commissioners of Health Visiting services, providers of Health Visiting and early years services, and members of the public.

Ethics

General University Ethics Panel approval has been obtained from University of Stirling (Reference 7662).

FUNDING STATEMENT

This study is funded by the NIHR Health and Social Care Delivery Research Programme NIHR134986. The views expressed are those of the author(s) and not necessarily those of the NIHR or the Department of Health and Social Care.

For peer review only

ACKNOWLEDGEMENTS

We wish to thank the members of our stakeholder and lived experience groups for their ongoing involvement in this study.

AUTHORS' CONTRIBUTIONS

Emma King – Methodology, Writing - Original Draft, Writing - Review & Editing,

Erica Gadsby – Conceptualisation, Methodology, Funding Acquisition, Supervision Writing - Review & Editing

Madeline Bell - Conceptualisation, Lived Experience Engagement, Writing - Review & Editing

Claire Duddy - Conceptualisation, Methodology, Funding Acquisition, Writing - Original Draft, Data Curation Writing - Review & Editing

Sally Kendall - Conceptualisation, Methodology, Funding Acquisition, Writing - Review & Editing

Geoff Wong - Conceptualisation, Methodology, Funding Acquisition, Writing - Review & Editing

COMPETING INTERESTS

None declared

FIGURES

For peer review only

Figure 1: Initial Programme Theory with possible contexts, mechanisms and outcomes of interest

For peer review only

REFERENCES

1. The Royal College of Nursing. The Best Start: The Future of

Children's Health – One Year On. RCN, Cavendish Square, London; 2018.
2. Hackett A, Clarke K, Wilkinson J. Social Policy, Context of Public Health and Organisation of Health Visiting Provision. In: Burrow P, Cowie J, editors. Health Visiting: Specialist Community Public Health Nursing 3rd ed Great Britain: Elsevier; 2022.
3. Whittaker K, Appleton, J.V., Peckover, S. and Adams, C. . Organising health visiting services in the UK: Frontline perspectives. . Journal of Health Visiting. 2021;9(2):68-75.
4. Cowley S, Whittaker K, Malone M, Donetto S, Grigulis A, Maben J. Why health visiting? Examining the potential public health benefits from health visiting practice within a universal service: a narrative review of the literature. Int J Nurs Stud. 2015;52(1):465-80.
5. Public Health England. Health visiting and school nursing service delivery model (updated 19 May 2021). Available: https://www.gov.uk/government/publications/commissioning-of-public-health-services-for-children/health-visiting-and-school-nursing-service-delivery-model (17/05/22); 2021.
6. Cowley S, Whittaker K, Malone M, Donetto S, Grigulis A, Maben J. Why health visiting? Examining the potential public health benefits from health visiting practice within a universal service: A narrative review of the literature. International Journal of Nursing Studies. 2015;52(1):465-80.
7. Axford N, Barlow, J., Coad, J., Schrader-Mcmillan, A., Bjornstad, G., Wrigley, Z., Goodwin, A., Ohlson, C., Sonthalia, S., Toft, A., Whybra, L. and Wilkinson, T. . Rapid Review to Update Evidence for the Healthy Child Programme 0–5. Available: https://assets.publishing.service.gov.uk/government/uploads/system/uploads/attachment_data/file/429740/150520RapidReviewHealthyChildProg_UPDATE_poisons_final.pdf (17/05/22); 2015.
8. Cowley S, Whittaker K, Malone M, Donetto S, Grigulis A, Maben J. What makes health visiting successful—or not? 1. Universality. Journal of Health Visiting. 2018;6(7):352-60.
9. Bunn F, Kendall S. Does nursing research impact on policy? A case study of health visiting research and UK health policy. Journal of Research in Nursing. 2011;16(2):169-91.
10. Conti G, Dow A. The impacts of COVID-19 on Health Visiting in England: First Results. Available: https://discovery.ucl.ac.uk/id/eprint/10106430 (17/05/22); 2020a.
11. Conti G, Dow A. The impacts of COVID-19 on Health Visiting Services in England: FOI Evidence for the First Wave. Available: https://discovery.ucl.ac.uk/id/eprint/10122752 (17/05/22); 2020b.
12. Saunders B, Hogg S. Babies in Lockdown: listening to parents to build back better. . Available: https://parentinfantfoundation.org.uk/our-work/campaigning/babies-in-lockdown/#fullreport (17/05/22); 2020.
13. Institute of Health Visiting. Making history: Health visiting during COVID-19. Available: https://ihv.org.uk/wp-content/uploads/2021/07/Health-visiting-making-history-case-studies-FINAL-VERSION-updated-14.7.21.pdf (17/05/22); 2020b.
14. Reed J, Parish N. Working for babies: Lockdown lessons from local systems. Available: https://parentinfantfoundation.org.uk/wp-content/uploads/2021/01/210115-F1001D-Working-for-Babies-Report-FINAL-v1.1-compressed-2.pdf (17/05/22); 2021.
15. Berlin LJ. Opening the black box: What makes early child and family development programs work. Zero to Three. 1998;18.4:1-40.

For peer review only

16. Gomby DS, Larson CS, Lewit EM, Behrman RE. Home visiting: Analysis and recommendations. The future of children. 1993:6-22.
17. Larner M. Inside the Black Box: Understanding Home Visiting Programs. 1987.
18. Lewis R, Pereira P, Thorlby R, Warburton W. Understanding and sustaining the health care service shifts accelerated by COVID-19. 2020.
19. Gadsby EW, Christie-de Jong, F., Bhopal, S., Corlett, H. and Turner, S. . Qualitative analysis of the impact of the SARS-CoV-2 pandemic response on paediatric health services in North of Scotland and North of England. BMJ Open. 2022;12(2).
20. Barlow J, Bach-Mortensen, A., Homonchuk, O. and Woodman, J. The impact of the COVID-19 pandemic on services from pregnancy through age 5 years for families who are high risk of poor outcomes or who have complex social needs. 2020.
21. Bynner C, McBride M. Children's Neighbourhood Scotland - Early insights into the COVID-19 response: 8 May 2020. . Available: http://eprints.gla.ac.uk/215811 (17/05/22). 2020.
22. Morton A. Health visiting during COVID-19 and beyond: the impact on children and families. Local Government Association webinar: Supporting the development of babies and young children during the COVID-19 outbreak, ; 2020.
23. Bryans A, Cornish, F. and McIntosh, J. . The potential of ecological theory for building an integrated framework to develop the public health contribution of health visiting. Health & Social Care in the Community. 2009;17(6):564-72.
24. Black MM, Walker, S.P., Fernald, L.C.H., Andersen, C.T., Digirolamo, A.M., Lu, C., Mccoy, D.C., Fink, G., Shawar, Y.R., Shiffman, P.J., Devercelli, A.E., Wodon, Q.T., Vargas-Barón, E. and Grantham-Mcgregor, S. . Advancing Early Childhood Development: from Science to Scale 1: Early childhood development coming of age: science through the life course. . The Lancet (British Edition). 2016;389(10064):77-90.
25. National Research Council, Institute of Medicine Committee on Integrating the Science of Early Childhood D. In: Shonkoff JP, Phillips DA, editors. From Neurons to Neighborhoods: The Science of Early Childhood Development. Washington (DC): National Academies Press (US); 2000.
26. HM Government. The Best Start for Life: A Vision for the 1,001 Critical Days. The Early Years Healthy Development Review Report CP419 (25 March 2021) Available: https://www.gov.uk/government/publications/the-best-start-for-life-a-vision-for-the-1001-critical-days (17/05/22); 2021.
27. Berg RC, Nanavati J. Realist Review: Current Practice and Future Prospects. Journal of Research Practice. 2016;12:1.
28. Duddy C, Wong G. Grand rounds in methodology: when are realist reviews useful, and what does a ‘good’ realist review look like? BMJ Quality & Safety. 2022:bmjqs-2022-015236.
29. Astbury B, Leeuw FL. Unpacking Black Boxes: Mechanisms and Theory Building in Evaluation. American Journal of Evaluation. 2010;31(3):363-81.
30. Concannon TW, Meissner P, Grunbaum JA, McElwee N, Guise JM, Santa J, et al. A new taxonomy for stakeholder engagement in patient-centered outcomes research. J Gen Intern Med. 2012;27(8):985-91.
31. Pawson R, Greenhalgh, T., Harvey, G. and Walshe, K. Realist synthesis - an introduction. London: ESRC; 2004.
32. Greenhalgh T, Peacock R. Effectiveness and efficiency of search methods in systematic reviews of complex evidence: audit of primary sources. BMJ. 2005;331(7524):1064-5.
33. Booth A, Clarke, M., Ghersi, D., Moher, D., Petticrew, M. and Stewart, L. . An international registry of systematic-review protocols. The Lancet (British Edition). 2011;377(9760):108-9.
34. Booth A, Briscoe S, Wright JM. The “realist search”: A systematic scoping review of current practice and reporting. Research Synthesis Methods. 2020;11(1):14-35.

For peer review only

For peer review only - http://bmjopen.bmj.com/site/about/guidelines.xhtml

35. Pawson R. Evidence-based policy: a realist perspective. London: Sage; 2011.
36. Wong G. Data gathering for realist reviews: Looking for needles in haystacks. In: Emmel N, Greenhalgh J, Manzano A, Monaghan M, Dalkin S, editors. Doing Realist Research. London: SAGE; 2018.
37. Pawson R, Tilley N. An introduction to scientific realist evaluation. Evaluation for the 21st century: A handbook: Sage Publications, Inc; 1997. p. 405-18.
38. Papoutsi C, Mattick, K., Pearson, M., Brennan, N., Briscoe, S. and Wong, G. Interventions to improve antimicrobial prescribing of doctors in training (IMPACT): a realist review. Health Services and Delivery Research. 2018;6(10).

For peer review only

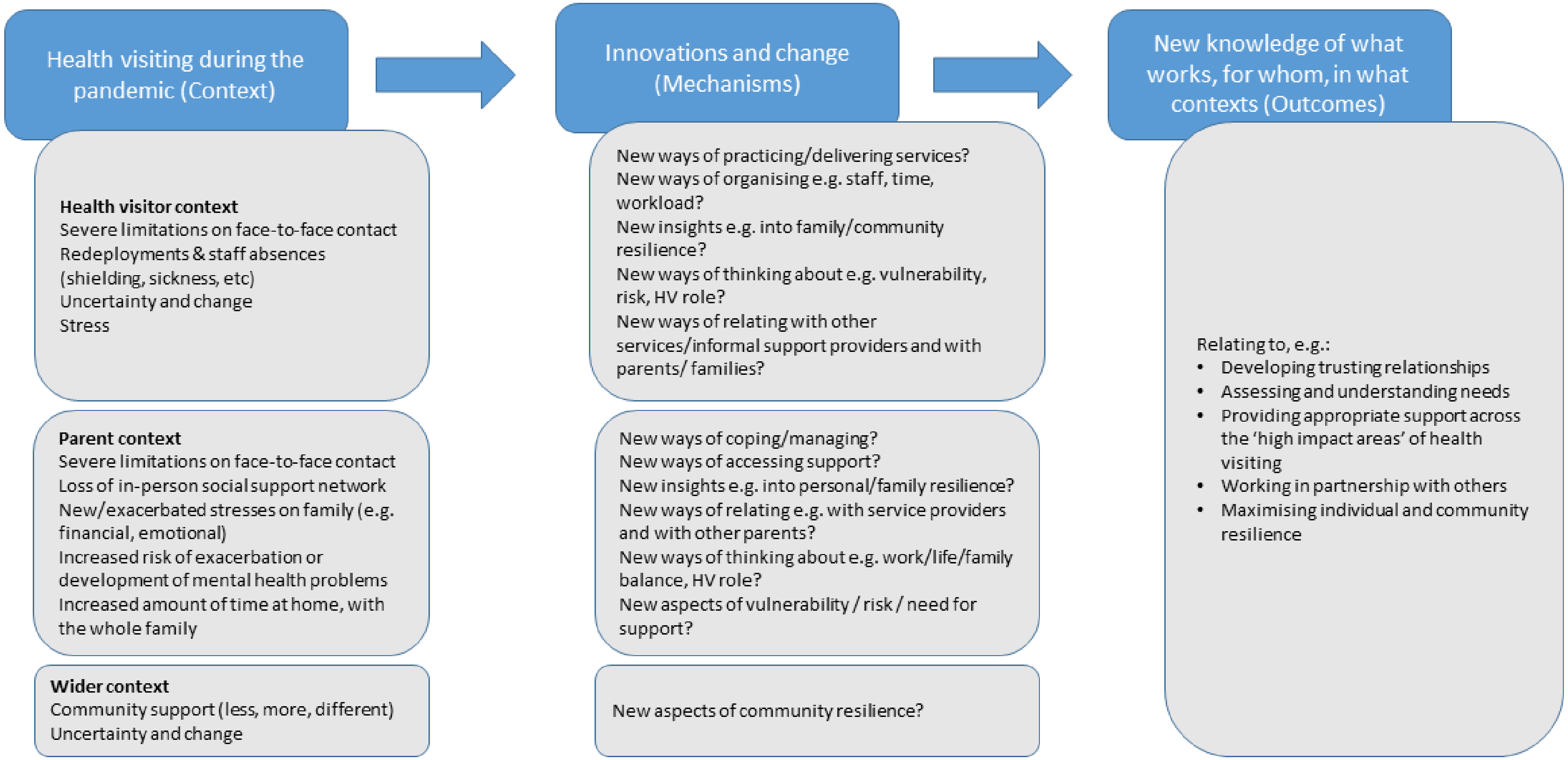
Health visiting during the pandemic (Context)
Innovations and change (Mechanisms)
New knowledge of what works, for whom, in what contexts (Outcomes)
Health visitor context
Severe limitations on face-to-face contact
Redeployments & staff absences (shielding, sickness, etc)
Uncertainty and change
Stress
Parent context
Severe limitations on face-to-face contact
Loss of in-person social support network
New/exacerbated stresses on family (e.g. financial, emotional)
Increased risk of exacerbation or development of mental health problems
Increased amount of time at home, with the whole family
Wider context
Community support (less, more, different)
Uncertainty and change
New ways of practicing/delivering services?
New ways of organising e.g. staff, time, workload?
New insights e.g. into family/community resilience?
New ways of thinking about e.g. vulnerability, risk, HV role?
New ways of relating with other services/informal support providers and with parents/ families?
New ways of coping/managing?
New ways of accessing support?
New insights e.g. into personal/family resilience?
New ways of relating e.g. with service providers and with other parents?
New ways of thinking about e.g. work/life/family balance, HV role?
New aspects of vulnerability / risk / need for support?
New aspects of community resilience?
Relating to, e.g.:
• Developing trusting relationships
• Assessing and understanding needs
• Providing appropriate support across the 'high impact areas' of health visiting
• Working in partnership with others
• Maximising individual and community resilience

Supplementary file 1

Search strategy for Ovid MEDLINE

MEDLINE® Epub Ahead of Print, In-Process & Other Non-Indexed Citations, Ovid MEDLINE® Daily and Ovid MEDLINE® 1946 to present

1	(health visitor* or health visiting).ti,ab,kw.
2	child health program*.ti,ab,kw.
3	healthy child program*.ti,ab,kw.
4	healthy child wales.ti,ab,kw.
5	getting it right for every child.ti,ab,kw.
6	healthy child healthy future.ti,ab,kw.
7	or/1-6
8	SARS-CoV-2/ or COVID-19/ or exp COVID-19 Testing/ or exp COVID-19 Vaccines/
9	(corona* adj1 (virus* or viral*)).ti,ab,kw,kf.
10	(coronavirus* or coronoravirus* or coronaravirus* or coronovirus* or 2019nCoV* or 19nCoV* or "2019 novel*" or Ncov* or "n-cov" or "SARSCoV-2*" or "SARSCoV-2*" or SARSCoV2* or "SARS-CoV2*" or "severe acute respiratory syndrome*" or COVID*2).ti,ab,kw,kf.
11	(covid* or pandemic).ti,ab,kw,kf.
12	(CoV not (Coefficien* or "co-efficien*" or covalent* or Covington* or covariant* or covarianc* or "cut-off value*" or "cutoff value*" or "cut-off volume*" or "cutoff volume*" or "combined optimi?ation value*" or "central vessel trunk*" or CoVR or CoVS)).ti,ab,kw,kf.
13	or/8-12
14	limit 13 to yr="2020-current"
15	7 and 14

The search strategy combines general terms describing health visitors and health visiting services, and terms referring to specific UK-based programmes and policies, with terms describing the Covid-19 pandemic. The latter set of terms were adapted from existing filters developed by the UK's National Institute of Care Excellence (NICE) and Health Security Agency (HSA). Searches are limited to identify literature published from 2020 onwards.

For peer review only

RAMESES II reporting standards for realist evaluations*

Protocol: Realist Review: Health visiting in light of the COVID-19 Pandemic Experience (RReHOPE)

	Reported in Document	Pages in Document
TITLE		
1. In the title, identify the document as a realist evaluation.	Y	1
SUMMARY OR ABSTRACT		
2. Journal articles will usually require an abstract, while reports and other forms of publication will usually benefit from a short summary. The abstract or summary should include brief details on: the policy, programme or initiative under evaluation; programme setting; purpose of the evaluation; evaluation question(s) and/or objective(s); evaluation strategy; data collection, documentation and analysis methods; key findings and conclusions Where journals require it and the nature of the study is appropriate, brief details of respondents to the evaluation and recruitment and sampling processes may also be included Sufficient detail should be provided to identify that a realist approach was used and that realist programme theory was developed and/or refined.	Y	1,2
INTRODUCTION		
3. Rationale for evaluation. Explain the purpose of the evaluation and the implications for its focus and design.	Y	3
4. Programme theory. Describe the initial programme theory (or theories) that underpin the programme, policy or initiative.	Y	4
5. Evaluation questions, objectives and focus. State the evaluation question(s) and specify the objectives for the evaluation. Describe whether and how the programme theory was used to define the scope and focus of the evaluation.	Y	3,4
6. Ethical approval. State whether the realist evaluation required and has gained ethical approval from the relevant authorities, providing details as appropriate. If ethical approval was deemed unnecessary, explain why.	Y	1
METHODS		
7. Rationale for using realist evaluation Explain why a realist evaluation approach was chosen and (if relevant) adapted.	Y	3
8. Environment surrounding the evaluation. Describe the environment in which the evaluation took place.	Y	2,3
9. Describe the programme policy, initiative or product evaluated. Provide relevant details on the programme, policy or initiative evaluated.	Y	2,3
10. Describe and justify the evaluation design. A description and justification of the evaluation design (i.e. the account of what was planned, done and why) should be included, at least in summary form or as an appendix, in the document which presents the main findings. If this is not done, the omission should be justified and a reference or link to the evaluation design given. It may also be useful to publish or make freely available (e.g. online on a website) any original evaluation design document or protocol, where they exist.	Y	4-6
11. Data collection methods. Describe and justify the data collection methods – which ones were used, why and how they fed into developing, supporting, refuting or refining programme theory. Provide details of the steps taken to enhance the trustworthiness of data collection and documentation.	Y	6
12. Recruitment process and sampling strategy. Describe how respondents to the evaluation were recruited or engaged and how the sample contributed to the development, support, refutation or refinement of programme theory.	N	n/a
13. Data analysis. Describe in detail how data were analysed. This section should include information on the constructs that were identified, the process of analysis, how the programme theory was further developed, supported, refuted and refined, and (where relevant) how analysis changed as the evaluation unfolded.	N	n/a
RESULTS		
14. Details of participants. Report (if applicable) who took part in the evaluation, the details of the data they provided and how the data was used to develop, support, refute or refine programme theory.	N	n/a
15. Main findings. Present the key findings, linking them to contexts, mechanisms and outcome configurations. Show how they were used to further develop, test or refine the programme theory.	N	n/a
DISCUSSION		
16. Summary of findings. Summarise the main findings with attention to the evaluation questions, purpose of the evaluation, programme theory and intended audience.	N	n/a
17. Strengths, limitations and future directions Discuss both the strengths of the evaluation and its limitations. These should include (but need not be limited to): (1) consideration of all the steps in the evaluation processes; and (2) comment on the adequacy, trustworthiness and value of the explanatory insights which emerged. In many evaluations, there will be an expectation to provide guidance on future directions for the programme, policy or initiative, its implementation and/or design. The particular implications arising from the realist nature of the findings should be reflected in these discussions	PARTLY	1, 7
18. Comparison with existing literature. Where appropriate, compare and contrast the evaluation's findings with the existing literature on similar programmes, policies or initiatives.	N	n/a
19. Conclusion and recommendations. List the main conclusions that are justified by the analyses of the data. If appropriate, offer recommendations consistent with a realist approach.	N	n/a
20. Funding and conflict of interest. State the funding source (if any) for the evaluation, the role played by the funder (if any) and any conflicts of interests.	Y	7

*Wong, G., Westhorp, G., Manzano, A. *et al.* RAMESES II reporting standards for realist evaluations. *BMC Med* **14**, 96 (2016). https://doi.org/10.1186/s12916-016-0643-1

For peer review only
